# Elevated NIBP/TRAPPC9 mediates tumorigenesis of cancer cells through NFκB signaling

**DOI:** 10.18632/oncotarget.3349

**Published:** 2015-01-31

**Authors:** Yonggang Zhang, Shu Liu, Hong Wang, Wensheng Yang, Fang Li, Fan Yang, Daohai Yu, Frederick V. Ramsey, George P. Tuszyski, Wenhui Hu

**Affiliations:** ^1^ Department of Neuroscience, Temple University School of Medicine, Philadelphia, PA, USA; ^2^ Department of Biotherapy, The Forth Affiliated Hospital, China Medical University, Shenyang, Liaoning, China; ^3^ Department of Clinical Sciences, Temple University School of Medicine, Philadelphia, PA, USA

**Keywords:** NFκB, TRAPPC9, trans-Golgi network, cancer cells, tumorigenesis

## Abstract

Regulatory mechanisms underlying constitutive and inducible NFκB activation in cancer remain largely unknown. Here we investigated whether a novel NIK- and IKK2-binding protein (NIBP) is required for maintaining malignancy of cancer cells in an NFκB-dependent manner. Real-time polymerase chain reaction analysis of a human cancer survey tissue-scan cDNA array, immunostaining of a human frozen tumor tissue array and immunoblotting of a high-density reverse-phase cancer protein lysate array showed that NIBP is extensively expressed in most tumor tissues, particularly in breast and colon cancer. Lentivirus-mediated NIBP shRNA knockdown significantly inhibited the growth/proliferation, invasion/migration, colony formation and xenograft tumorigenesis of breast (MDA-MB-231) or colon (HCT116) cancer cells. NIBP overexpression in HCT116 cells promoted cell proliferation, migration and colony formation. Mechanistically, NIBP knockdown in cancer cells inhibited cytokine-induced activation of NFκB luciferase reporter, thus sensitizing the cells to TNFα-induced apoptosis. Endogenous NIBP bound specifically to the phosphorylated IKK2 in a TNFα-dependent manner. NIBP knockdown transiently attenuated TNFα-stimulated phosphorylation of IKK2/p65 and degradation of IκBα. In contrast, NIBP overexpression enhanced TNFα-induced NFκB activation, thus inhibiting constitutive and TNFα-induced apoptosis. Collectively, our data identified important roles of NIBP in promoting tumorigenesis via NFκΒ signaling, spotlighting NIBP as a promising target in cancer therapeutic intervention.

## INTRODUCTION

Nuclear Factor kappa B (NFκB) as a transcriptional factor can be activated by many extracellular cues, leading to expression of a variety of target genes important in such biological processes as inflammation, immunity, neural plasticity and pathological conditions including cancer and chronic inflammatory diseases [1-3]. Elevated basal activity of NFκB is found in many types of human cancer, especially in breast cancer. Sustained activation of NFκB induced by inflammatory mediators is critical for inflammation-related cancer [4, 5]. NFκB signaling has long been targeted for cancer therapy [6-12]. However, the clinical efficacy remains limited.

The canonical and non-canonical pathways for NFκB activation have been identified [1]. The canonical cascade is triggered by classical stimuli, such as tumor necrosis factor α (TNFα) and interleukin(IL)-1β, and depends on the formation of signalsome of IκB kinases (IKK1-IKK2-IKKγ). IKK2 phosphorylates inhibitory protein of NFκB (IκB) to target it for ubiquitination and degradation. This results in the nuclear translocation of NFκB heterodimers (mainly p65/p50) and the expression of target genes. The non-canonical pathway relies on phosphorylation of IKK1 by NFκB-inducing kinase (NIK) to induce limited proteasome-mediated processing of p100 and the nuclear translocation of RelB/p52 heterodimers. In order to identify novel regulators in NFκB signaling, we previously performed a yeast two-hybrid assay using NIK as bait and identified a novel protein called NIK- and IKK2-Binding Protein (NIBP). We demonstrated that NIBP enhances the activation of cytokine-induced NFκB signaling [13]. This characteristic function of NIBP was confirmed by two recent studies [14, 15]. Early studies demonstrated that almost all inducers of NFκB lead to activation of the canonical IKK1/2/γ complex. Recent studies identified additional IKK subcomplex, such as NIK/IKK1 [16, 17] and IKK2/IKKγ [18]. NIK/IKK1 predominantly mediates the non-canonical NFκB pathway while IKK2/IKKγ subcomplex remains functional for the canonical NFκB pathway. Our studies demonstrated that a novel subcomplex NIK-NIBP-IKK2 without IKK1 and IKKγ exists in neurons and brain tissue [13]. However, the functional relevance of the novel protein NIBP and the NIK-NIBP-IKK2 subcomplex [13] remains to be determined. In addition, the cellular specificity of NFκB signaling and the regulatory mechanisms underlying IKK2/NFκB activation remain elusive.

NIBP was recently renamed as trafficking protein particle complex 9 (TRAPPC9) because it is identical to yeast Trs120 protein, an essential and unique subunit for the TRAPP II complex that regulates the *trans*-Golgi exit process [19-22]. The *trans*-Golgi network is essential for cancer development and may represent a novel target for anti-cancer therapy [23]. TRAPPC10 and other TRAPP II-specific subunits are required for the transport of autophagy proteins from the *trans*-Golgi to a phagophore assembly site [24]. Autophagy is also shown to be a key mediator for tumorigenesis [25, 26]. Therefore, NIBP may regulate the *trans*-Golgi function and autophagosome formation during cancer development.

To understand whether NIBP and its regulated NFκB signaling are implicated in tumorigenesis, we performed bioinformatics evaluation on NIBP expression in different databases including Unigene, ACEVIEW, and Gene Expression Omnibus. We found that NIBP transcripts are present in various cancer cell lines and tumor tissues, but the correlation results are inconsistent between different databases. Clinical studies using high-density gene microarray and high-throughput functional screen reported a potential correlation of NIBP transcripts with human breast [27] and colon cancer [28], osteosarcoma [29], and lymphoma [30, 31]. In this study, we systematically characterized the expression profile of NIBP in various tumor biopsies and cancer cell lines by quantitative PCR (qPCR) analysis of a human cancer survey tissue-scan cDNA array, immunostaining of a human frozen tumor tissue array and immunoblotting of a high-density reverse-phase cancer protein lysate array. We also investigated potential roles of NIBP in regulating NFκB signaling, growth/proliferation, apoptosis, invasion/migration and tumor formation of human breast and colon cancer cells.

## RESULTS

### NIBP is highly expressed in human cancer cell lines and tumor tissues

To validate the NIBP expression in cancer cells, we performed Northern blot with a probe specific to a fragment harboring 1640-2423 bp in the reported NIBP cDNA clone (NM_031466). This probe detected NIBP transcript as a single band near 4.3 kb in selected cancer cell lines from breast (MCF7), cervix (HeLa) and gut (AGS, HCT116, Caco-2). Such transcript was undetectable by Northern blot in HEK293T and smooth muscle cells (Fig. S1A). Further reverse transcription (RT)-qPCR analysis using a pair of primers covering 771-914 bp of NM_031466 (primer pair 2) showed that NIBP is at high transcriptional levels in breast (MCF7) and gut cancer cell lines (Fig. S1B). Consistently, RT-qPCR analysis with another pair of primers covering 3122-3266 bp of NM_031466 (primer pair 1) identified high levels of NIBP transcripts in the selected cancer cell lines (Fig. S1B). These data support that NIBP are highly expressed in human breast and gut cancer cell lines, in accord with bioinformatics information collected from some database searching.

**Figure 1:**
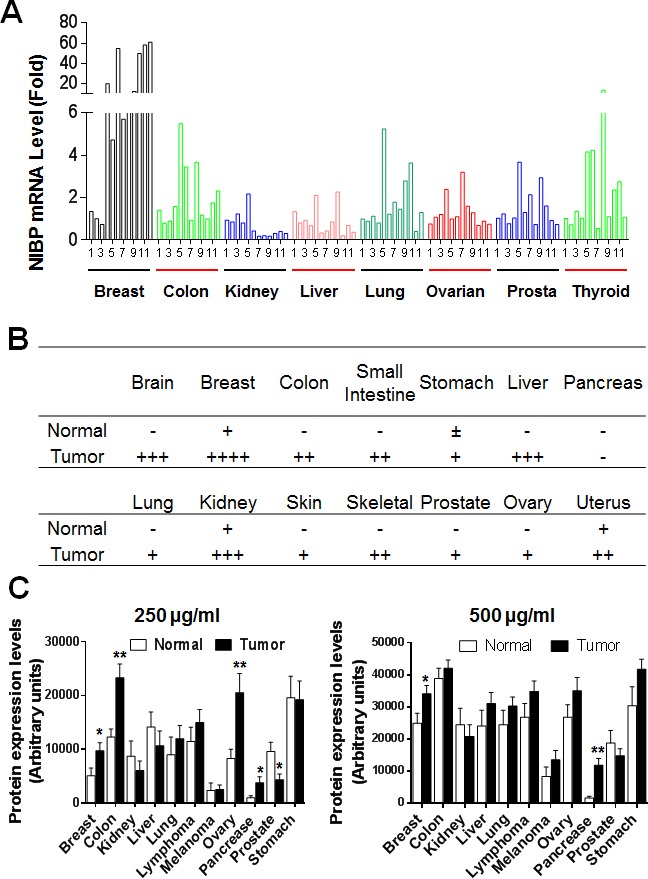
NIBP expression is upregulated in most tumor tissues (A) A TissueScan cancer survey qPCR analysis identified the increased expression of NIBP mRNA in most tumor tissues. The data represent fold changes of NIBP mRNA expression in a tumor sample to a mean value of the corresponding normal tissues after β-actin normalization. (B) Semi-quantitative evaluation of NIBP-like immunoreactivity in the frozen tissue microarray from indicated cancer patients showed dramatic increases in NIBP protein expression in most tumor tissues as compared to corresponding normal tissues. A 5-grade scoring method was employed on the basis of the area and positivity of immunofluorescent staining. (C) A high-density reverse-phase cancer protein lysate array was evaluated for NIBP protein expression in 11 tumor tissues at various concentrations (250 and 500 μg/ml) of loaded protein lysates. * p<0.05 and ** P<0.01 indicates significant difference between tumor samples and corresponding normal tissues using Student's *t* test.

To ultimately define the expression profile of NIBP in human tumor tissues, we performed qPCR analysis of a human cancer survey tissue-scan cDNA array (CSRT501, Origene), immunostaining of a human frozen tumor tissue array (T6235700, AMSBIO) and immunoblotting of a high-density reverse-phase cancer protein lysate array (PA100002, Origene). Extensive expression of NIBP mRNA was detected in tumor tissues from the indicated organs, wherein the highest expression level was found in breast tumor, and significant up-regulation was observed in colon and thyroid tumors (Fig. 1A). Limited up-regulation of NIBP mRNA was revealed in kidney and liver tumor tissues, perhaps due to a higher expression of NIBP mRNA in their corresponding normal tissues (Fig. S2A). Further analysis of the human breast tissue from the cancer survey cDNA array indicated that the NIBP mRNA expression in nearly half of breast cancer tissues increased approximately by 50-fold though the case number was limited (Fig. 1A, S2B). Immunohistochemical staining of the case-limited frozen tissue microarray showed extensive NIBP-like immunoreactivity in different tumor tissues as compared with corresponding normal tissues, with most obviously positive staining in tumors from breast, liver, brain and kidney (Fig. 1B, S3), but relatively lower staining signals in gastrointestinal (GI)/reproductive system, skeleton, skin and lung. Immunoblotting studies on the cancer protein array of 431 specimens from 25 tumor samples and 15 normal samples per type of 11 human tissues with 5 dilutions of protein lysate in triplicates, and corresponding cancer cell lines demonstrated a significantly increased expression of NIBP protein in most organ tumors (Fig. 1C, S4A-C) and cancer cell lines (Fig. S4D) as compared with corresponding normal tissues. The levels of statistical significance varied with the amount of loading protein in different organ tissues, indicating a variation of the immunoblotting detection sensitivity. Reduced NIBP expression was observed in kidney and prostate tumor tissues (Fig. 1C, S4C). Taken together, our data provided convincing evidence that NIBP is highly up-regulated at both mRNA and protein levels in the majority of human cancer cells and tumor tissues.

### Elevated NIBP promotes the proliferation and colony formation of cancer cells

**Figure 2:**
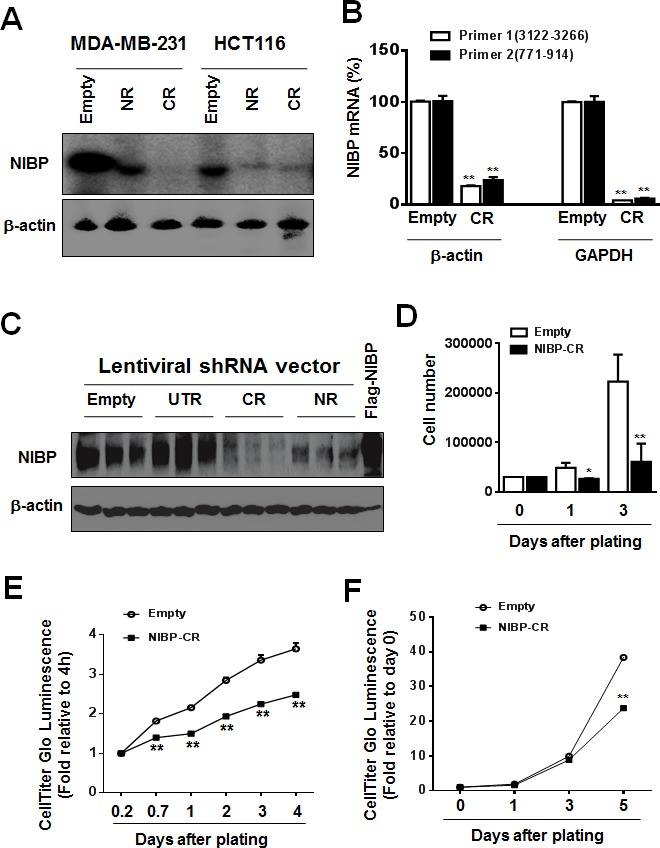
NIBP knockdown by lentivirus-mediated shRNAs inhibits cancer cell growth/proliferation (A-C) The efficacy of NIBP knockdown in cancer cells was validated in cancer cells. The MDA-MB-231 (A) or HCT116 (A-C) cells were transduced with indicated lentiviral vectors encoding shRNA targeting 5′-coding region (NR), 3′-coding region (CR) and 3′-untranslated (UTR) regions of human NIBP. After cell sorting with an internal GFP marker and passaging four times, the levels of NIBP mRNA (A, B) and protein (C) were determined by Northern blot (A), RT-qPCR (B) and immunoblotting analyses (C). The β-actin or GAPDH was used for loading control. The pRK-Flag-NIBP transfected cells were used as a positive control for immunoblotting. (D-F) Hemocytometry (D) and Cell-Titer Glo luminescence viability assays (E, F) showed significant inhibition of cell growth in MDA-MB-231(D, E) and HCT116 (F) cells at passage 4. ** P<0.01 indicates a significant decrease in time-dependent viability/proliferation of NIBP-CR shRNA knockdown cells as compared with corresponding empty vector controls.

To determine the biological relevance of highly expressed NIBP in breast and colon cancer cells/tissues, we established lentivirus-mediated NIBP stable knockdown tumor cell lines. Four short hairpin RNAs (shRNAs) encoded by 4 different regions targeting the 5′-(NR), 3′-coding region (CR) and 3′-untranslated regions (UTR) of human NIBP (NM_031466) were designed for the cloning into lentiviral shRNA expression vector pLL3.7 and their efficacies were evaluated as we described previously [13, 32]. Using the most effective shRNAs, NIBP-NR and -CR [13], we established breast (MDA-MB-231) and colon (HCT116) cancer cell lines with NIBP stable knockdown. Cell sorting using an internal GFP marker was performed to enrich lentivirus-infected cells for each cell line. The efficacy of shRNA-induced NIBP knockdown in cancer cells was further validated by Northern blot, RT-qPCR analysis and immunoblotting (Fig. 2A-C). The most effective NIBP-CR shRNA was hereafter used in our present studies. The empty pLL3.7 lentiviral vector and the ineffective NIBP-UTR lentiviral vector were used as negative controls.

**Figure 3:**
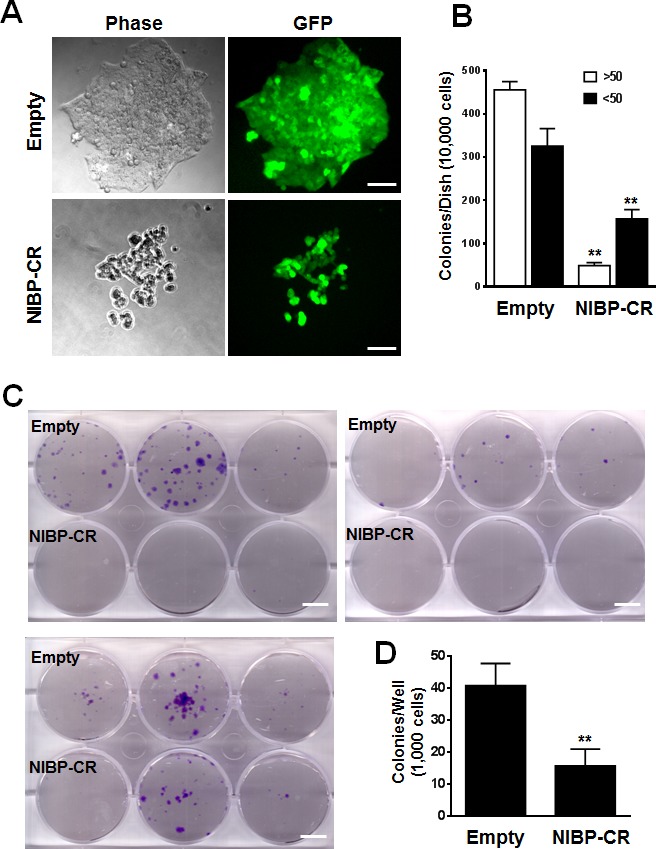
Lentivirus-mediated shRNA knockdown of NIBP inhibits the colony formation of cancer cells *in vitro* (A, B) Anchorage-dependent colony formation was significantly inhibited in NIBP-CR shRNA transduced HCT116 cells (passage 5). An equal number of exponentially growing cancer cells with or without stable NIBP shRNA knockdown were seeded in a 6-well tissue culture plate and incubated for 2 weeks. All colonies with at least 10 cells were counted under a standard inverted fluorescent microscope. The values (B) represent mean ± SD of three independent experiments each with triplicate wells. (C, D) Anchorage-independent growth was significantly reduced in NIBP-CR shRNA transduced MDA-MB-231 cells (passage 4). Equal numbers of cancer cells were resuspended in 1 ml of 0.3% top agar and plated on 2 ml of 0.8% bottom agar in each well of a 6-well plate. After 3 weeks, cell colonies were visualized by crystal violet staining (C) and quantified by counting the colony number (D). ** p<0.01 indicates a significant decrease in NIBP-CR shRNA knockdown as compared with corresponding empty vector control. Scale bar = 50 μm (A) or 10 mm (C).

To examine the effects of endogenous NIBP knockdown on the proliferation and viability of cancer cells, we performed Trypan blue staining and CellTiter-Glo(R) luminescent cell viability assay. NIBP knockdown significantly inhibited cell proliferation and viability in MDA-MB-231 (Fig. 2D, E) and HCT116 (Fig. 2F). To test if high levels of endogenous NIBP expression in cancer cells promote the colony formation, a distinctive characteristic of tumorigenesis, we performed colony formation assays in an anchorage-dependent (Fig. 3A, B) or -independent manner (Fig. 3C, D). The colony formation was significantly reduced in both breast and colon cancer cell lines after lentivirus-mediated stable NIBP knockdown (Fig. 3A-D). These data suggest that NIBP is required for the proliferation and colony formation of cancer cells from breast and colon.

To further examine the role of NIBP in regulating the proliferation and colony formation of cancer cells, we overexpressed NIBP in HCT116 cells using the lentivirus-mediated gene delivery system. Overexpression of Flag-tagged NIBP was validated by immunocytochemistry and immunoblotting with anti-Flag antibody. NIBP overexpression induced a marginal but significant increase in the cell proliferation/viability (Fig. S5A, B) and colony formation (Fig. S5C, D). The marginal effect may be attributed to the higher levels of endogenous NIBP expression in cancer cells.

### Elevated NIBP promotes the invasion/migration of cancer cells

**Figure 4:**
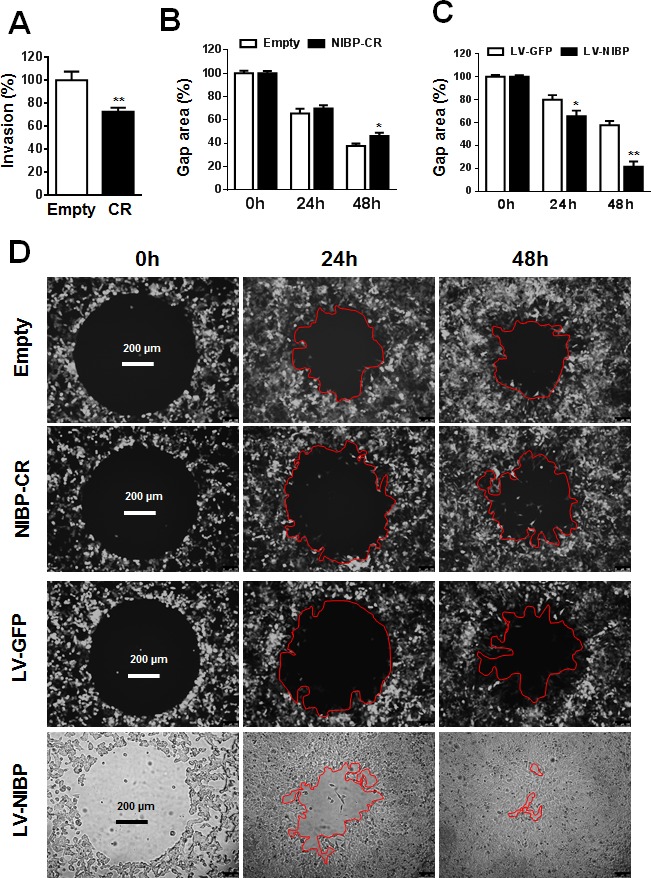
NIBP promotes cancer cell invasion/migration (A) Lentivirus-mediated NIBP knockdown significantly inhibited the invasion of HCT116 cells as determined by the Boyden chamber assay. Equal numbers of cells (300,000 per well) were seeded into polycarbonate membrane inserts in a 24-transwell culture plate. After 24 h incubation, cells having invaded the membrane were extracted for spectrophotometric measurement (560 nm). Values represent percentage change over 100% arbitrarily set for the empty vector control. (B-D) NIBP knockdown inhibited (B) while NIBP overexpression promoted (C) the migration of HCT116 cells as determined by a gap closure assay. Equal number of cells (10,000 per well) were seeded at 10 wells each group in a 384-well plate. A circular gap was generated by removing a biocompatible hydrogel in each well. At 24 and 48 h, the gap area (%) was measured by micrograph and image analysis. Representative micrographs were shown in D. * p<0.05 and ** p<0.01 indicates significant changes in the NIBP knockdown or overexpression group as compared with the corresponding empty control group.

Invasion/migration capability is a characteristic feature for cancer cells leading to tumor metastasis. To determine the biological effect of NIBP knockdown or overexpression on cell invasion/migration, we performed the Boyden chamber assay using a trans-well CytoSelect™ cell invasion assay kit and a gap closure assay using a newly developed Radius™ cell migration assay kit. The number of HCT116 cells invading through the basal membrane into the lower chamber was significantly reduced by 30% in stable NIBP knockdown cells compared with empty lentiviral control cells (Fig. 4A). The Radius™ technology integrates each well in a 384-well plate a biocompatible hydrogel to create a circular gap area across which cells can migrate. As shown in Fig. 4B-D, HCT116 cancer cells migrated to close the gap in a time-dependent manner. NIBP knockdown reduced the migration (gap closure) at 24 and 48 h after gel removal with a statistical significance at 48 h. In contrast, NIBP overexpression dramatically increased the migration at both 24 and 48 h with a statistical significance. These data suggest that NIBP is required for the migration of cancer cells.

### Stable knockdown of NIBP inhibits tumor formation of cancer cells in nude mice

**Figure 5:**
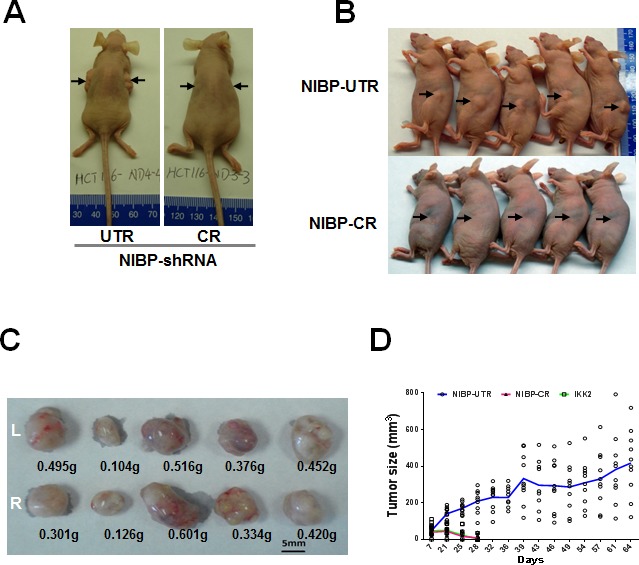
Lentivirus-mediated shRNA knockdown of NIBP inhibits the xenograft tumor formation of HCT116 (A) and MDA-MB-231 (B-D) cancer cells in nude mice Equal numbers of exponentially growing cells (1×10^7^ cells per site) stably expressing indicated shRNAs were mixed in matrigel and injected subcutaneously into the left and right flanks of female nu/J mice (n=5). Each tumor was measured twice a week for 9 weeks. A, B, Representative pictures taken at 8 weeks after injection showing tumor/injection sites (arrow). C, Tumor weight at the end of experiments showing the tumors growing at the left (L) and right (R) flanks of animals injected with NIBP-UTR lentivirus. D, The tumor growth curve for the ineffective NIBP-UTR group, the effective NIBP-CR group and the IKK2-shRNA group.

To determine the role of NIBP in regulating the tumor growth of MDA-MB-231 and HCT116 cells *in vivo*, we injected equal number of cancer cells subcutaneously into both flanks of female nude mice. Due to slower growth, the NIBP stable knockdown cells were passaged in a higher density during *in vitro* culture to reach equal numbers of cancer cells for injection. The shRNA empty or NIBP-ineffective (UTR) lentiviral vector transduced cells were used as negative controls and IKK2-shRNA lentiviral vector [33] transduced cells as a positive control. Xenograft growth in mice was examined twice a week for 2-3 months. In the NIBP-ineffective control group, the tumor grew in 1-2 weeks from all of the injection sites and continued growing until the mice were euthanized (Fig. 5). Comprehensive pathology examination at euthanization did not identify any tumors in other skin regions and organs in all groups of animals. In the NIBP-effective shRNA group and IKK2-shRNA group, tumors grew in 1-2 weeks from 20-30% of injection sites, but stopped growing after 2-3 weeks, and finally no tumor was detected at 3 months. These distinct phenotypic results suggest that stable knockdown of NIBP significantly block tumor formation of human breast and colon cancer cells in immune-deficient mice, hence placing NIBP as an important regulator in human cancer.

### NIBP up-regulates NFκB signaling in cancer cells

**Figure 6:**
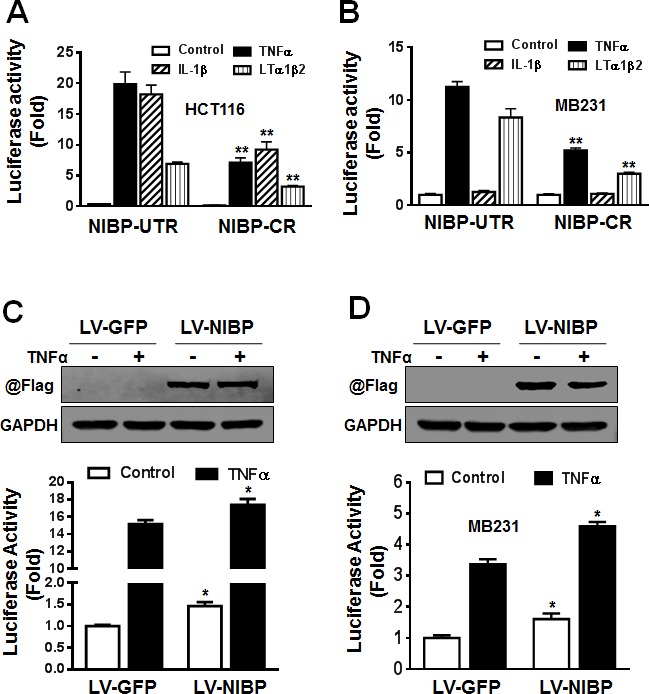
NIBP enhances NFκB activation in cancer cells (A, B) Stable NIBP knockdown significantly inhibited cytokine-stimulated NFκB activation in HCT116 (A) and MDA-MB-231 (B) cells. The cancer cells at passage 5 were infected with adenovirus carrying an NFκB-*firefly*-luciferase reporter. After 24 h, cells were serum-starved overnight and treated with indicated cytokines for 24 h before GloOne luminescence assay. (C, D) Lentivirus (LV)-mediated overexpression of NIBP induced constitutive and TNFα-stimulated NFκB activation in HCT116 (C) and MDA-MB231 (D) cells. Cells were infected with indicated lentiviruses followed by an adenovirus-mediated NFκB *firefly*-luciferase reporter assay. The Flag-tagged NIBP expression was validated by immunoblotting with anti-Flag antibody and anti-GAPDH antibody used as loading control. The data represent mean ± SD of 3-5 independent experiments. * p<0.05 and ** p<0.01 indicates significant changes in the NIBP knockdown or overexpression group as compared with empty control lentiviral vectors.

We demonstrated previously that NIBP enhanced TNFα- and IL-1β-induced activation of the NFκB canonical pathway as determined by the NFκB-driven reporter assay, gel shift assay and immunoblotting analysis for cytokine-induced IκBα degradation and IKK2/p65 phosphorylation [13]. Three recent studies added to our finding that NIBP increased TNFα-induced NFκB activation in HEK293T cells, fibroblasts, and neurons [32, 34, 35]. Since NIBP was highly expressed in cancer cells and NFκB/IKK2 signaling is critical for tumorigenesis, we thus investigated whether NIBP affects NFκB signaling in cancer cells. The results from NFκB-luciferase reporter assay revealed that the effective NIBP-CR shRNA significantly impaired TNFα-induced activation of NFκB reporter in HCT116 cells (Fig. 6A) and MDA-MB-231 cells (Fig. 6B). NIBP knockdown also significantly inhibited lymphotoxin (LT) α1/β2-induced NFκB activation in both cell lines (Fig. 6A, B). However, IL-1β stimulated NFκB reporter activation in HCT116 cells, which was significantly inhibited by NIBP-CR shRNA (Fig. 6A), but exerted no effect on NFκB activation in MDA-MB-231 cells (Fig. 6B). Consistently, overexpression of NIBP induced constitutive activation of NFκB signaling and enhanced TNFα-stimulated NFκB activation in HCT116 (Fig. 6C) and MDA-MB-231 cells (Fig. 6D). These data suggest that NIBP modulates the constitutive and inducible activation of NFκB signaling in breast and colon cancer cells.

**Figure 7:**
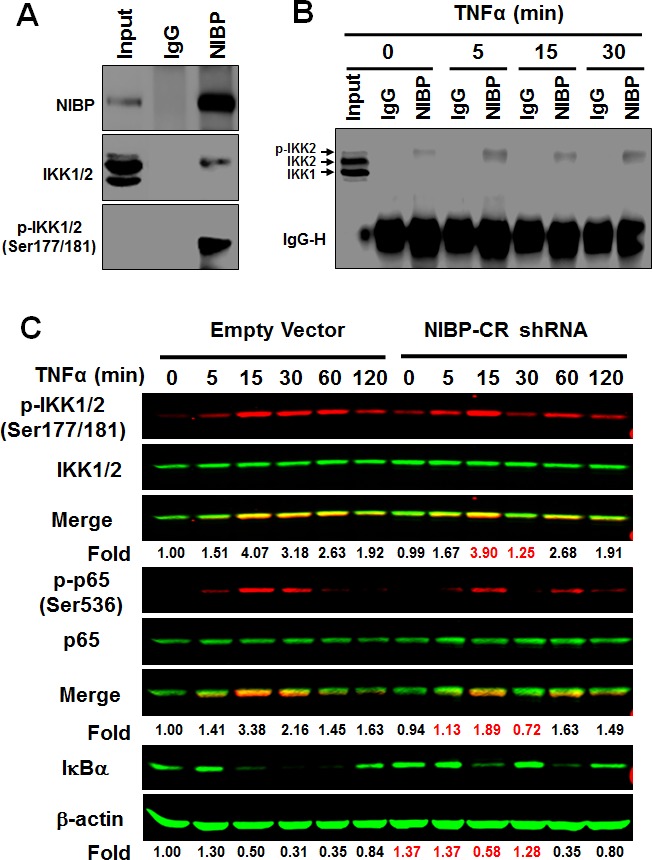
NIBP interacts with phosphorylated IKK2 and modulates TNFα-induced activation of the classical IKK2/IκBα/p65 pathway in cancer cells (A, B) Endogenous NIBP was coimmunoprecipated with phosphorylated IKK2 in HCT116 cells. Cells were treated with TNFα for 5-30 min and whole cell lysates were immunoprecipitated with anti-NIBP antibody or control IgG followed by immunoblotting with antibodies against NIBP (A), IKK1/2 (A, B) or phosphorylated IKK1/2 (A). Input accounts for 1% of used lysates. (C) TNFα-induced activation of the classical IKK2/IκBα/p65 pathway in MDA-MB-231 cells was inhibited by stable NIBP knockdown at 5-30 min. Cells were infected with lentiviruses carrying empty vector or NIBP-CR shRNA and sorted by FACS. After 5 passages, equal numbers of cells in 6-well plates were treated with TNFα for the indicated time intervals and the expression of indicated proteins was analyzed by immunoblotting using the Odyssey CLx Infrared Fluorescent Immunoblotting system. The signal intensities were determined using the LI-COR imaging software and the numbers below the blot indicate relative fold changes compared to empty vector vehicle control after normalization by total IKK1/2, p65 or β-actin respectively.

In HEK293T cells, PC12 cells, enteric neuronal cell lines and brain tissues, NIBP has been shown to interact with IKK2 but not IKK1 [13, 32]. Such interaction contributes to the enhancing effect of NIBP on cytokine-induced activation of the NFκB canonical pathway [13]. To determine if endogenous NIBP also interacts with IKK2 in cancer cells, we performed co-immunoprecipitation experiments in HCT116 cells. Interestingly, only phosphorylated IKK2 was co-immunoprecipitated with NIBP as demonstrated by the presence of band shift in comparison with that of the input (Fig. 7A, B) and the specific recognition with anti-phospho-IKK1/2(Ser-177/181) antibody (Fig. 7A). This observation is consistent with our previous report that NIBP enhances IKK2 kinase activity by increasing stimulatory phosphorylation of IKK2 [13]. Such interaction occurred dynamically, being increased at 5 min, recovered at 15 min and increased again at 30 min after TNFα treatment (Fig. 7B). Furthermore, a significant reduction of the endogenous NIBP expression in cancer cells by lentivirus-mediated NIBP shRNA inhibited TNFα-induced phosphorylation of IKK1/2 and p65, and degradation of IκBα at 5-30 min of TNFα treatment with most dramatic inhibition at 30 min (Fig. 7C). In the untreated group, the IκBα level was higher in NIBP-CR cells than that in the control cells, indicating that NIBP knockdown also inhibited constitutive degradation of IκBα and thus activation of NFκB. These data suggest that NIBP is required for TNFα-induced activation of the canonical IKK2/IκBα/p65 signaling pathway in cancer cells but the modulation is transient.

### NIBP/NFκB signaling maintains the survival of cancer cells

**Figure 8:**
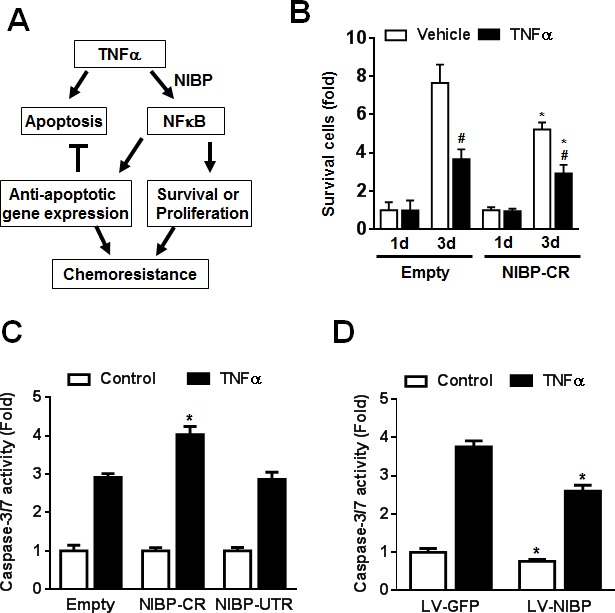
NIBP maintains cancer cell survival (A) Schematic representation of the cancer cell NIBP/NFκB-dependent regulation of chemoresistance. (B) TNFα-induced cell death in HCT116 cells with NIBP knockdown was examined. An equal number of cells (10,000 cells/well) were seeded in 96-well plates. After 24 h, cells were treated with or without TNFα (10 ng/ml) for 1 and 3 d and the viable cell number was determined by trypan blue staining and hemocytometry. The data represent fold change related to the corresponding vehicle control at 1 d. (C) NIBP shRNA knockdown sensitized TNFα-induced apoptosis in HCT116 cells. Equal numbers of cells stably engineered with indicated shRNA were cultured in a 96-well plate (5,000 cells per well in quadruplicate) and treated with TNFα (10 ng/ml) for 24 h before *Caspase-Glo® 3/7 assay was performed.* (D) NIBP overexpression inhibited constitutive and TNFα-induced apoptosis in HCT116 cells. Cells were infected with indicated lentivirus (LV) and the third passage cells (5,000 cells per well in quadruplicate) were treated with TNF (10 ng/ml) for 24 h before *Caspase-Glo® 3/7 assay was performed.* * P<0.05 indicates a significant difference compared with the corresponding empty lentiviral vector.

TNFα stimulation of cancer cells activates the caspase-dependent apoptotic pathway and the NFκB-dependent cell survival pathway that contributes to the chemoresistance or radiation-resistance of cancer cells (Fig. 8A) [36, 37]. As expected, inhibition of TNFα-induced NFκB activation by NIBP-CR shRNA in HCT116 cells significantly sensitized the cells to TNFα-induced apoptosis as determined by cell survival (Fig. 8B) and the activity of caspase-3/7 (Fig. 8C). In contrast, NIBP overexpression in HCT116 cells inhibited constitutive and TNFα-induced apoptosis (Fig. 8D). Taken together, NIBP-mediated NFκB activation plays an important role in proliferation and survival of cancer cells in response to extracellular insult.

## DISCUSSION

NIBP represents a novel family of proteins according to the PANTHER classification system. A decade of studies has identified the important functions of NIBP in regulating NFκB signaling [13-15, 32], *trans*-Golgi networking [19-22] and virus replication [35]. In the present study, we demonstrated that NIBP is highly expressed in numerous cancer cell lines and tumor tissues, and provided evidence for the first time that NIBP plays an important role in regulating viability, proliferation, migration and tumorigenesis of breast or colon cancer cells, notably in response to extracellular pro-inflammatory stimuli. NIBP may serve as a novel biomarker for predicting prognosis and a therapeutic target for the diagnosis and treatment of cancer, particularly the breast and colon cancer.

NFκB is a ubiquitous transcriptional factor and plays a pivotal role in a number of pathophysiological processes including inflammation, immune response, aberrant cell growth or apoptosis [2, 3]. All these processes, when deregulated, acquire mechanisms to drive cancer initiation and progression. High constitutive expression of NFκB signaling components, particularly the IKK complex, is present in cancer cells but not in normal cells, suggesting the important role of NFκB and IKK in regulating tumorigenesis [4, 5, 38-48]. Sustained activation of NFκB is a critical mechanism of inflammation-linked cancer [4, 5, 48]. IKK2 is essential in cancer metastasis [49, 50] and tumorigenesis [51, 52]. In breast cancers, IKK2 overexpression is associated with cytoplasmic accumulation of p21, an antiapoptotic factor involved in tumorigenesis [53]. Therefore, suppression of NFκB and especially IKK2 activation has been targeted for drug development and clinical application [6-12]. However, mechanisms underlying constitutive and inducible activations of NFκB and IKK2 in cancer remain largely unclear. Our previous studies showed that a novel regulator of NFκB signaling, NIBP binds to IKK2/NIK and enhances cytokine-induced NFκB activation [13]. NIBP increases the kinase activity of IKK2 [13]. In the present study, we demonstrate that (a) NIBP mRNA and protein were highly expressed in many cancer cell lines and tumor tissues; (b) NIBP knockdown inhibited the proliferation, migration and colony formation of breast and colon cancer cell lines *in vitro* as well as the tumor formation *in vivo*; (c) NIBP overexpression promoted cell survival, migration and colony formation; (d) NIBP maintains high levels of constitutive NFκB activity and enhances cytokine-induced NFκB activation through the classical IKK2/IκBα/p65 pathway. These findings support an imminent conclusion that endogenous NIBP aberrantly expressed in cancer cells may trigger and retain constitutive activity and enhance inducible activation of IKK2/NFκB signaling, as a result contributing to the tumorigenesis and cancer metastasis. Noticeably, NIBP transiently and dynamically up-regulates TNFα-induced activation of the classical NFκB pathway, supporting the concept that NIBP acts as an adaptor protein to modulate NFκB signaling [13-15]. Elegantly designed mouse models with NIBP gene knockout or knockin are underway to trace the NIBP roles in tumorigenesis. Furthermore, the case number for the survey/screening studies on NIBP expression patterns in various tumor tissues was limited. A large cohort of clinical studies on NIBP expression in different types of cancer are being processed and conclusive results will be pursued to establish NIBP as a novel biomarker and target for the prognosis and therapy of human cancer.

In cancer cells, NFκB signaling is activated by different inflammatory cytokines. TNFα is deemed consistently to stimulate NFκB activation in different cancer cells. LT-α1β2 is also well known to stimulate NFκB activation in most cells [54-56]. Interestingly, we found that IL-1β, a common stimulator for NFκB signaling in most cells, fails to activate NFκB in MDA-MB-231 cells other than in HCT116 cells. This finding may provide an interpretation to the previous report that NFκB signaling is not involved in the IL-1β-induced upregulation of Cox2 expression in MDA-MB231 cells where Cox2 is an NFκB target gene [57, 58]. Thus various cytokines regulate NFκB signaling in a cell-specific manner.

Our previous studies showed that NIBP also interacts with NIK, a key mediator for the non-canonical NFκB pathway. Further investigation is warranted to determine whether NIBP affects non-canonical NFκB pathway. Recent studies have demonstrated that NIBP is a key subunit specific to the complex II of trafficking protein particles (TRAPP), designated TRAPPC9 [19-22] and regulates *trans*-Golgi network by interacting with other subunits of the TRAPP-II complex, such as TRAPPCs 2, 3, 4, 8, 10 and 12 [19-22]. However, the biological relevance of NIBP/TRAPPC9 in the *trans*-Golgi network and protein trafficking remains to be determined [19].

In summary, NIBP/TRAPPC9 is highly expressed in cancer cell lines and tumor tissues. High levels of NIBP expression may induce the deregulated cell proliferation, thus promoting colony formation *in vitro* and tumor formation *in vivo*. NIBP/TRAPPC9 enhances the basal activity and cytokine-induced activation of the classical NFκB signaling. Our understanding of NIBP/TRAPPC9 functions in cancer particularly in breast/colon cancer from the present study proposes that targeting NIBP/TRAPPC9 should witness a novel strategy for clinical diagnosis and therapeutic intervention to better identify and treat cancer patients.

## MATERIALS AND METHODS

### Cell culture and transfection

The cells including HEK293T, MCF7, MD-MB231, HCT116, AGS, Caco-2, Hela, NIH3T3, THP-1S, H-SY5Y and NT2 were purchased from the American Type Culture Collection and grown in high-glucose DMEM containing 10% FBS and antibiotics (100 U/ml penicillin and 100 μg/ml streptomycin) in a humidified atmosphere with 5% CO2 at 37 °C. The human colonic smooth muscle cell line was kindly provided by Dr. Karnam S. Murthy at Virginia Commonwealth University. All cells were verified being free of mycoplasma contamination. For immunoblotting and immunoprecipitation experiments, cells were seeded in 10 cm dishes. For colony formation assay, cells were seeded in 6-well plates. For cell-based functional assays, cells were seeded in 96-well plates. All transfections were performed with lipofectamine 2000 according to the manufacturer's manual.

### Lentivirus-mediated NIBP knockdown or overexpression

Lentiviral vectors encoding enhanced green fluorescent protein (GFP) cloned with DNA inserts expressing shRNA targeting the 5′-coding region (NR, 5′-gagattgatgtacacagcattc-3′), 3′-coding region (CR, 5′-caaagttgaacttgtccac-3′) and 3′-untranslated region (UTR, 5′-gcaacataaatacacagac-3′) of human NIBP transcripts as well as three regions of human IKK2 mRNAs were generated using a modified PCR-based strategy as previously described [13, 59]. Lentiviral vector encoding mouse NIBP (960 aa) was generated by cloning NIBP (960 aa) PCR fragments (coding sequence) into a pRRL-GFP vector through *SalI/SpeI* digestion sites as described previously [60, 61].

Packaging, purification and titer determination of the lentiviruses were performed as described previously [62, 63]. All recombinant lentiviruses were produced from HEK293T cells after calcium phosphate-mediated transient transfection of related vectors according to standard protocols. Briefly, HEK293T cells were co-transfected with the lentiviral transfer vector (10 μg), lentiviral packaging vectors pRSV-REV (2 μg) and pMDLg/pRRE (5 μg), and vesicular stomatitis virus G glycoprotein (VSVG) expression vector pMD2G (3 μg). The viruses were collected from the culture supernatant on days 2 and 3 post-transfection, concentrated by ultracentrifugation for 1.5 h at 25,000 rpm, and then resuspended in phosphate-buffered saline (PBS). Virus titer determination was performed by infecting HEK293T cells with serial diluted lentiviruses and counting the number of GFP-expressing cells 48 h post-transfection under fluorescent microscopy. For a typical preparation, the titer was approximately 4−10×10^8^ infectious units per ml. For lentiviral infection of cancer cells, the cells in a 6-well plate were cultured in medium and inoculated with lentivirus at a multiplicity of infection (MOI) of 50 for 24 h. The percentage of cells that became infected at the MOI was approximately 95%. To enrich NIBP-shRNA positive cells and then obtain the stable NIBP-knockdown cell lines, fluorescence activated cell sorting (FACS) was performed at a core facility.

### Quantitative reverse transcription-polymerase chain reaction (RT-qPCR)

For total RNA extraction, cells were processed with an RNeasy Mini kit (Qiagen) as per manufacturer's instructions. The potentially residual genomic DNA was removed through on-column DNase digestion with an RNase-Free DNase Set (Qiagen). One μg of RNA for each sample was reversely transcribed into cDNAs using random hexanucleotide primers with a High Capacity cDNA Reverse Transcription Kit (Invitrogen, Grand Island, NY). Quantitative PCR (qPCR) analyses were carried out in a LightCycler480 (Roche) using an SYBR® Green PCR Master Mix Kit (Applied Biosystems) as described previously [64]. The RT reactions were diluted to 5 ng of total RNA per micro-liter of reactions and 2 μl was used in a 20-μl PCR reaction. The two pairs of human NIBP primers each targeted the range 3122-3266 bp (primer pair #1) or 771-914 bp (primer pair #2) of human NIBP cDNA (NM_031466). The primers for human housekeep genes GAPDH and β-actin were obtained from RealTimePrimers (Elkins Park, PA). Each sample was tested in triplicate. Cycle threshold (Ct) values were obtained graphically for the target genes and house-keeping genes. The difference in Ct values between the housekeep gene and target gene were represented as ΔCt values. The ΔΔCt values were obtained by subtracting the ΔCt values of control samples from those of experimental samples. Relative fold change in gene expression was calculated as 2-ΔΔCt. In some cases (Figure S1B), absolute quantification was performed using NIBP plasmid as a standard.

### Northern blot

The equal amount of total RNA (10 μg) extracted from cells was used for Northern blot according to a standard protocol. The RNA blots were hybridized with a PCR-produced, [^32^P]dCTP-labeled 780-bp probe in the ExpressHyb hybridization solution (Clontech) at 65°C for 2 h according to the manufacturer's protocol. The PCR probe for NIBP was generated using a pair of primers targeting 1640-2423 bp of human NIBP (NM_031466). Primers for 18s were forward GTTGGTGGAGCGATTTGTCT and reverse GGCCTCACTAAACCATCCAA covering 1345-1739 bp of human 18s rRNA (NR_003286.2).

### TissueScan qPCR profile of human cancers

The TissueScan Cancer Survey qPCR arrays (CSRT501) containing cDNAs from 8 different types of cancer (breast, colon, kidney, liver, lung, ovarian, prostate and thyroid) were purchased from Origene Technologies (Rockville, MD, USA). Primers for human NIBP (primer pair #2) covering 771-914 bp of the cDNA sequence (NM_031466) and human β-actin were used for SYBR® Green qPCR as described above. The results were represented from three PCR assays.

### Immunohistochemical staining of human frozen tumor tissue microarray

The frozen tumor and normal multi-tissue array mounted on slides (T6235700, lot# A803046, AMSBIO) was fixed with 4% paraformaldehyde for 30 min, permeated for 30 min in PBS containing 0.5% Triton X-100 and blocked with 10% normal donkey serum for 30 min. The slide was incubated with affinity-purified rabbit anti-NIBP (417) antibody at 1:500 in PBS with 0.1% Triton X-100 for 1 d at 4°C. After washing three times each for 10 min, the slide was incubated in Alexa Fluor®-488 conjugated donkey anti-rabbit secondary antibody (1:400; Invitrogen, Grand Island, NY) for 1 h at room temperature. The specificity of anti-NIBP antibody was tested as described previously [13, 32]. Hoechst 33258 was used for nuclear counterstaining. The tissue array was coverslipped with anti-fading aqueous mounting media. The immunofluorescent staining was visualized under a fluorescent inverted microscope (Nikon, Japan) equipped with a cooled CCD camera using Slidebook 5.0 digital imaging software. For each spot, 3 micrographs were taken under a 20x objective. The staining intensity and positive area within each spot were scored using a 5-grade evaluation system (− to ++++).

### Immunoblotting and immunoprecipitation

Cells were lysed in Triton X-100-based lysis buffer (20 mM Tris-HCl (pH 7.4), 1% Triton X-100, 5 mM ethylenediaminetetraacetic acid, 5 mM dithiothreitol, 150 mM NaCl, 1 mM phenylmethylsulfonyl fluoride, 1x nuclear extraction proteinase inhibitor cocktail (Cayman Chemical, Ann Arbor, MI), 1 mM sodium orthovanadate and 30 mM NaF). Lysates were rotated at 4°C for 30 min. Nuclear and cellular debris in lysates was removed by centrifugation at 20,000 g for 20 min at 4°C. The protein concentration of supernatant was determined using a Pierce BCA Protein Assay Kit (cat# 23225). An equal amount of protein lysate (20 μg) was denatured by heating for 5 min in sodium dodecyl sulphate (SDS) sample buffer, resolved by the SDS-polyacrylamide gel electrophoresis system using tris-glycine running buffer, and proteins in PAGE gels were transferred to nitrocellulose (NC) membrane (BioRad). The SeeBlue prestained protein standards (Invitrogen) were used as a molecular weight reference. NC membrane blots were blocked in 5% nonfat dry milk dissolved in tris-buffered saline (pH 7.6) plus 0.1% Tween-20 (TBS-T) for 1 h and then incubated overnight at 4°C with the affinity-purified rabbit anti-NIBP polyclonal antibodies diluted at 1:3,000, designated anti-NIBP(417) that was generated using the peptide 417-VYNPMPFELRVENMGLLTSGVEF-439 in mouse NIBP(960), which is 100% homologous to that in human, rat and rabbit as described previously [13, 32]. Other primary antibodies for the immunoblotting were as follows: p-IKK1/2 (1:1,000), p-p65 (1:2,000) and IκBα (1:1,000) from Cell Signal Technology; IKK1/2 (1:2,000) and p65 (1:2,000) from Santa Cruz Biotechnology; and β-actin (1:10,000) from Sigma. After incubation for 1 h with corresponding horseradish peroxidase-conjugated secondary antibodies (1/2000; 10 μg/ml, Pierce) in TBS-T containing 1% milk, immune-reactive proteins were visualized using a SuperSignal Femto maximum sensitivity substrate kit (Thermo Fisher Scientific, Rockford, IL). All washing steps were performed using TBS-T. In some cases, the Odyssey CLx Infrared Fluorescent Immunoblotting system (LI-COR, Lincoln, NE) was used according to the manufacture's instruction. Briefly, NC membranes were blocked with Odyssey blocking buffer containing 0.1% (v/v) Tween 20. The membranes were incubated overnight at 4°C with rabbit and mouse primary antibodies and then washed several times followed by incubation with fluorescently conjugated secondary antibodies (IRDye 680LT-conjugated anti-mouse or IRDye 800CW-conjugated anti-rabbit) for 1 h at room temperature. The membranes were scanned and analyzed using the Odyssey Infrared Imaging System. Relative signal intensities were determined using the LI-COR imaging software.

For immunoprecipitation, precleared cell lysates (300-500 μg) were incubated overnight at 4°C with 0.5 μg of the anti-NIBP antibody or the rabbit IgG control. The immune complexes were precipitated with protein A/G plus agarose beads, and the beads were washed four times using high salt lysis buffer (containing 0.6 M NaCl). Immunoprecipitated proteins were eluted in the SDS sample buffer and denatured by boiling for 5 min. The eluted proteins were fractionated in the SDS-polyacrylamide gel electrophoresis and detected by immunoblotting using indicated antibodies.

### Immunoblotting of cancer protein lysate array

A large-scale reverse phase ProteoScan Cancer Lysate Array 2.0 from OriGene (PA100002) contains 431 protein lysates of normal and cancer specimens derived from 11 different tissues. Bio-specimens were obtained from accredited academic and medical institutions in the United States. Samples and data were collected strictly according to the IRB and HIPAA guidelines. Tissues were removed from the protective OCT by incubation in TBS buffer containing a protease inhibitor cocktail (Sigma P2714). The samples were homogenized and extracted in modified RIPA buffer (50 mM Tris-Hcl pH 7.5, 150 mM NaCl, 1 mM EDTA, 1% NP40 and 0.25% Deoxy-cholate) containing the protease inhibitor cocktail (Sigma P2714), 0.4 mM PefaBlock SC plus (Roche 11873601001) and a PhosStop phosphatase inhibitor cocktail (Roche 04906845001). Following extraction the lysates were centrifuged to remove the insoluble and stored at −80°C. Protein concentrations were determined by the BCA assay. All lysates were adjusted to 1 mg/ml using modified RIPA buffer and diluted to 500, 250, 125 and 62.5 μg /ml in RIPA buffer. The arrays were printed on Grace-Bio lab SuperNova nitrocellulose slides using non-contact and inkjet printing technologies. The total volume of an aliquot deposited within each spot was approximately 300 pl. The quality of each print was verified by Sypro-ruby protein staining. A sample was printed in triplicate each located on a different sub-array to ensure accuracy and reproducibility. The Proteoscan cancer lysate array 2.0 contains 33 sub-arrays each with the configuration of 15 × 16 spots. Positive placement controls (BSA-Cy3, BSA-Cy5 and the mixture of human, mouse and rabbit IgGs) were placed in each sub-array. BSA background (negative) controls at 1,000, 500, 250, 125 and 62.5 μg/ml were also placed in each sub-array. The IgG mixtures (human, mouse and rabbit) were used as positive controls with 8 diluted concentrations (10-0.078 μg/ml) and a group of purified recombinant β-actin at five diluted concentrations (10-0.3125 μg/ml) was placed twice on the array. Cancer cell lines of the corresponding tissue were chosen from the NCI60 cancer cell lines list and the cell lysate was printed on sub-arrays related to 7 tissues with a concentration similar to the tissue lysate. The representative array layout and sample location were shown in the Fig. S3A.

The ProteoScan Cancer Lysate Array was immunoblotted using the Odyssey CLx Infrared Fluorescent Immunoblotting system as described above. Briefly, the array was probed with the rabbit anti-NIBP (417) polyclonal antibody (1:3,000) followed by IRDye 800CW-conjugated anti-rabbit secondary antibody. The arrays were scanned using the Odyssey Infrared Imaging System. The images were exported as tiff files and analyzed with GenePix Pro software (Origene). The cancer expression index is calculated by dividing the readout from each cancer cell sample with an expression median from the normal samples of the same tissue. Further statistical analysis was performed in a biostatistics core.

### NFκB-luciferase reporter assay

Lentivirus-infected stable cells were cultured in a 96-well plate and infected with adenovirus expressing the NFκB-driven *firefly*-luciferase reporter (Vector Biolabs) at an MOI of 100 for 24 h. The cells were serum-starved for 24 h before they were treated with or without indicated cytokines for 24 h. To examine *firefly* luciferase activity, the cell lysate was prepared using the ONE-Glo luciferase assay system (Promega) and luminescence was measured in a 2104 EnVision® Multilabel Reader (PerkinElmer). Representative results were obtained from three separate experiments each of which generated a result calculated as a mean of values from 4-6 samples.

### Cell growth/proliferation assay

The cell growth/proliferation was determined by the trypan blue exclusion hemocytometry, and CellTiter-Glo luminescence viability assay (Promega). The CellTiter-Glo luminescent cell viability assay is a homogeneous and sensitive method to quantitate ATP generated by metabolically active cells that associates with the number of viable cells. Briefly, cells were cultured in sterile 96-well plates in the presence or absence of indicated reagents for 1-5 d and then 100 μl of CellTiter-Glo reagent was used to lyse the cells. After incubation for 10 min at room temperature, the luminescence in each well was measured in a 2104 EnVision® Multilabel Reader (PerkinElmer). Data were generated accordingly as described above.

### Anchorage-dependent and independent colony formation assay

To determine effects of NIBP shRNA knockdown on the colony formation of cancer cells, the standard anchorage-dependent colony formation assay and soft agarose assay were performed as described previously [65, 66]. In an anchorage-dependent assay, an equal amount of MDA-MB231 cells (1,000 cells/well) or HCT116 cells (10,000 cells/well) was seeded in 6-well tissue culture plates and cultured for 2 weeks. The number of colonies with at least 10 cells per well was counted under an inverted fluorescent microscope using a 10x objective. For an anchorage-independent soft agarose assay, the equal number of MDA-MB231 cells (2,000 cells/well) was suspended in 1 ml of 0.3% Noble Agar (Sigma) dissolved in the complete culture medium (DMEM with 10% FBS and 1% penicillin/streptomycin) and plated on the top of the pre-made bottom agar (2 ml of 0.6% agar in the same medium in the 6-well culture plates). Three weeks after incubation in a humidified incubator with 5% CO2 at 37°C, cell colonies were fixed with methanol and stained with 0.5% crystal violet for 1 hour, photographed and counted under a regular light microscope.

### CytoSelect™ cell invasion assay

The ability of HCT116 cells to pass through basal membrane was measured with a CytoSelect™ cell invasion assay kit (CBA-110, Cell Biolabs). Briefly, cells after overnight serum starvation were harvested, washed, and suspended in serum-free culture media. Equal numbers of cells (300,000 per well) were seeded into polycarbonate membrane inserts (8 μm pore size) in a 24-transwell culture plate. The lower chamber was filled with the complete culture medium (DMEM with 10% FBS and 1% penicillin/streptomycin). After 24 h incubation in a humidified incubator with 5% CO2 at 37°C, the cells in the upper surface of the membrane were carefully removed with a cotton swab. Cells invading the membrane to the lower surface of the membrane were extracted with the extraction solution and an optical density at 560 nm was measured in a plate reader.

### Radius™ cell migration assay

The migration ability of HCT116 cells was measured with a gap closure assay using a Radius™ cell migration assay kit (CBA-127, Cell Biolabs). Equal number of cells (10,000 per well) were seeded in 10 wells each group in a 384-well plate. When cells were confluent, the RADIUS gel spot was removed according to the manufacturer's protocol in order to simulate a wound and the cells were cultured for 2 days. The pre-migration and daily post-migration images were captured using an invert fluorescent microscope DMI6000B (Leica). Areas were measured using the “outline” function on the Image J program. The percentage of gap areas over time was calculated by dividing the original RADIUS open.

### Cell apoptosis assay

HCT116 cells were infected with lentiviruses expressing NIBP-targeting (NIBP-CR) or control shRNAs (empty vector or UTR), or NIBP (GFP as a control) and several passages passed before cells were seeded in a 96-well plate. In each well, 5,000 cells were growing overnight and then treated with human TNFα at 10 ng/ml for 24 h before caspase-3/7 activities were examined using a Caspase-Glo® 3/7 Assay (Promega, Madison, WI) according to the product manual.

### Xenograft tumor formation model

The breast (MDA-MB-231) and colon (HCT116) tumorigenic cells were infected with related shRNA-expressing lentiviruses and infected cells were enriched by FACS. Before injection, the efficiency of shRNA knockdown was further validated by RT-qPCR. The cells (1 × 10^7^) stably engineered with lentiviral shRNA vectors were mixed with matrigel (BD Biosciences) and injected subcutaneously into the flank of athymic nude mice (Nu/J, female, 5-6 weeks old, Jackson Lab). For each cell line, the animals were randomly grouped and injected with the cells stably integrated with empty lentiviral vector (pLL3.7), effective NIBP shRNA vector, ineffective NIBP shRNA vector or effective IKK2 shRNA vector respectively. Five animals per group were used as described previously [67-70]. The size of tumor mass and mouse weight were recorded twice a week for two months and the tumor volume was calculated (by the equation volume=width^2^ x length x 0.5). At the end of the experiment the animals were euthanized with CO_2_ inhalation before the tumor masses were weighed. Animal treatment and maintenance were conducted in accordance with guidelines approved by the Temple University Institutional Animal Care and Use Committee (IACUC).

### Statistical analysis

GraphPad Prism 6 software was used for statistical analysis. The data were presented as mean ± standard deviation from 3-5 independent experiments, and analyzed using Student's *t*-test or ANOVA and Newman-Keuls multiple comparison test. The comparison with a p value < 0.05 (*) or 0.01 (**) was considered as a statistically significant difference.

## SUPPLEMENTARY MATERIAL, FIGURES


