# Supplementary material for: Elevated NIBP/TRAPPC9 mediates tumorigenesis of cancer cells through NFκB signaling
Source: Oncotarget. 2015 Jan 31;6(8):6160–78. doi: 10.18632/oncotarget.3349 (PMC4467429; doi:10.18632/oncotarget.3349)
Supplement: Supplementary file 1 [file oncotarget-06-6160-s001.pdf]

Elevated NIBP/TRAPPC9 mediates tumorigenesis of cancer cells through NFκB signaling

Supplementary Material

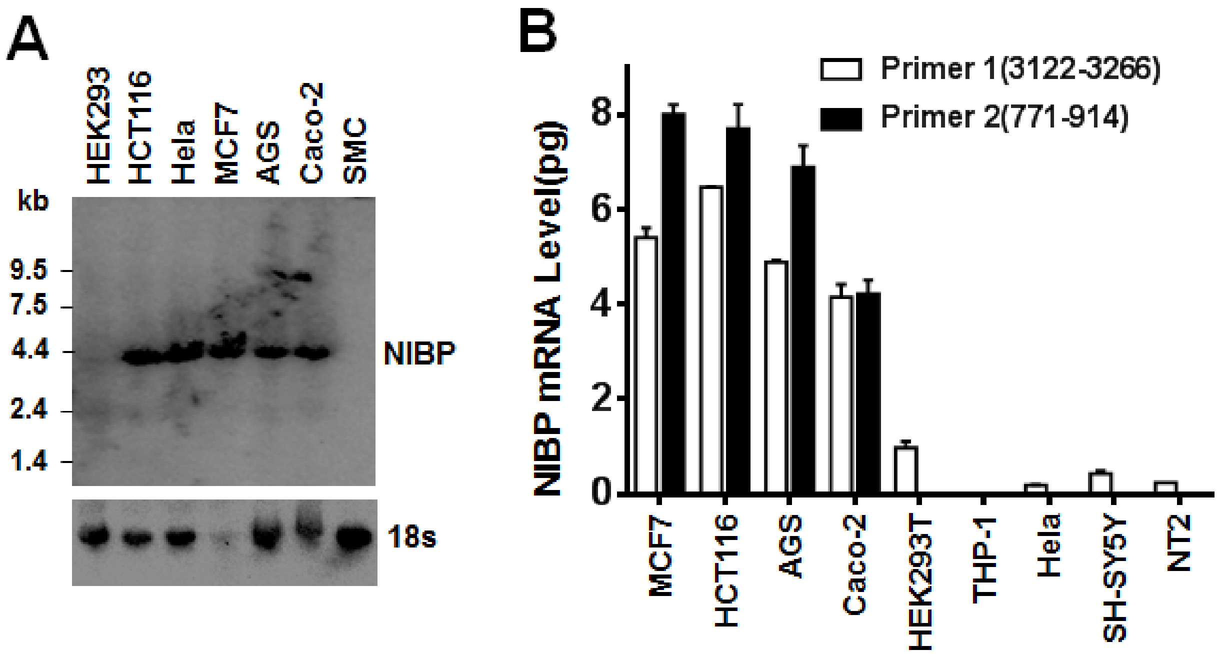

Figure S1: NIBP mRNA expression is upregulated in cancer cell lines. (A) Northern blot analysis detected a single transcript highly expressed in cancer cells. The equal amounts of total RNA (10 μg) from indicated cells were subject to Northern blotting with a radiolabeled NIBP probe and 18s rRNA was used as a loading control. SMC, primary colonic smooth muscle cells. (B) The levels of NIBP mRNA expression in indicated cell lines were assessed by RT-qPCR analysis. Two different pairs of primers showed similar upregulation of NIBP mRNA expression in breast and gut cancer cells.

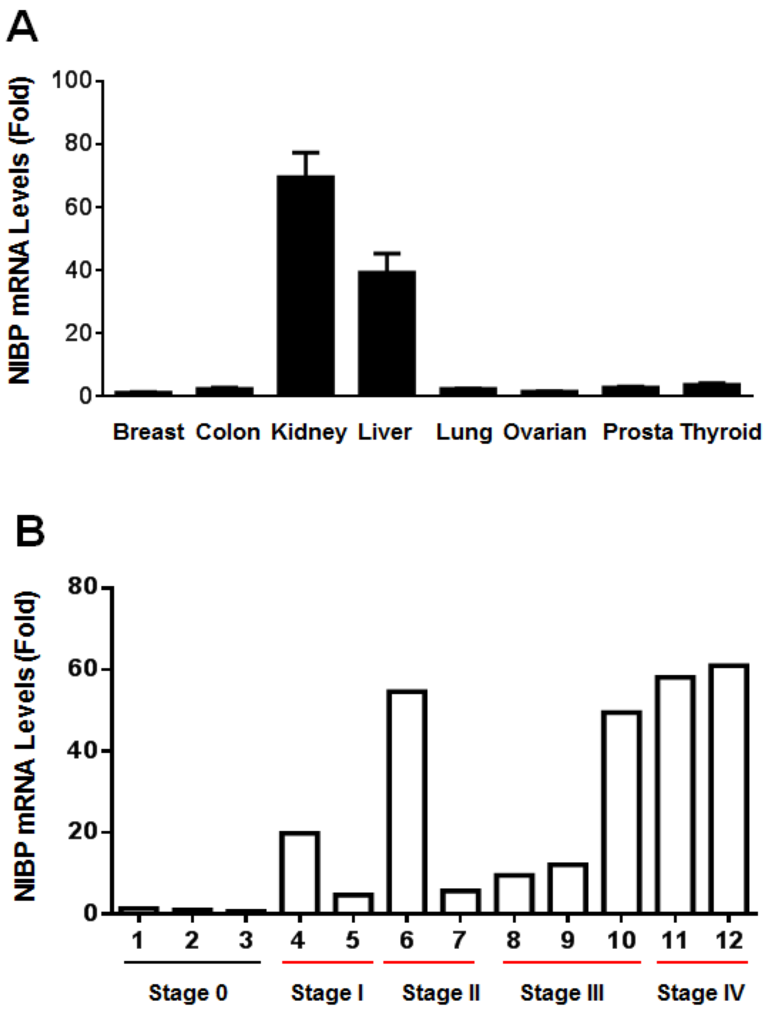

Figure S2: TissueScan cancer survey qPCR analysis shows relative expression levels of NIBP mRNA in human normal tissues (A, n=3) and representative change in different stage of breast (B) cancer tissues. The data represent fold change relative to the average level in the breast tissues (A) or in the corresponding normal tissues (B) after β-actin normalization.

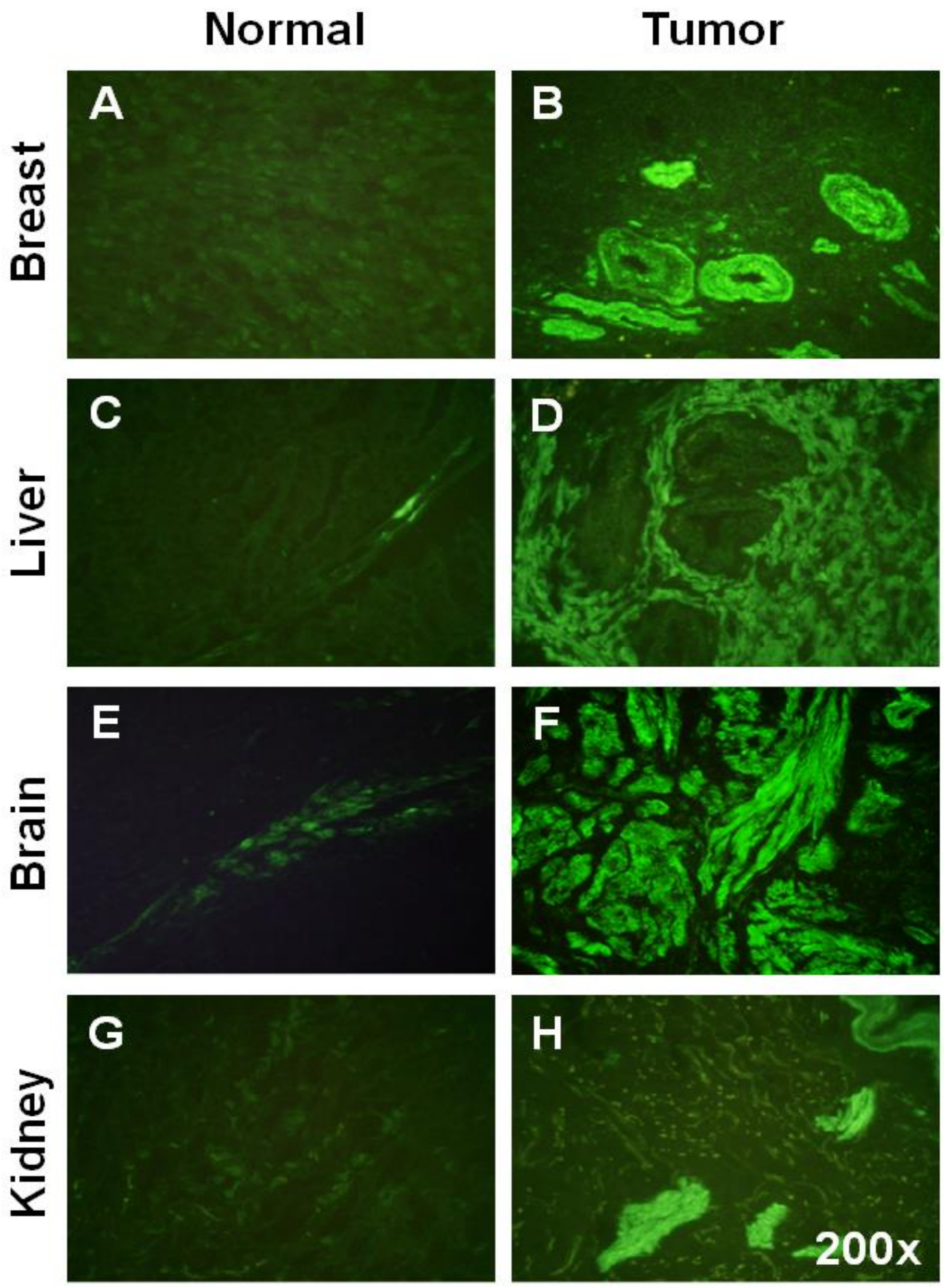

Figure S3: Representative micrographs of NIBP-like immunoreactivity in normal and tumor tissues from indicated human organs. A frozen tissue microarray (T6235700, AMSBIO) was immunostained with anti-NIBP antibody (1:500) and NIBP protein expression was evaluated using fluorescent microscopy.

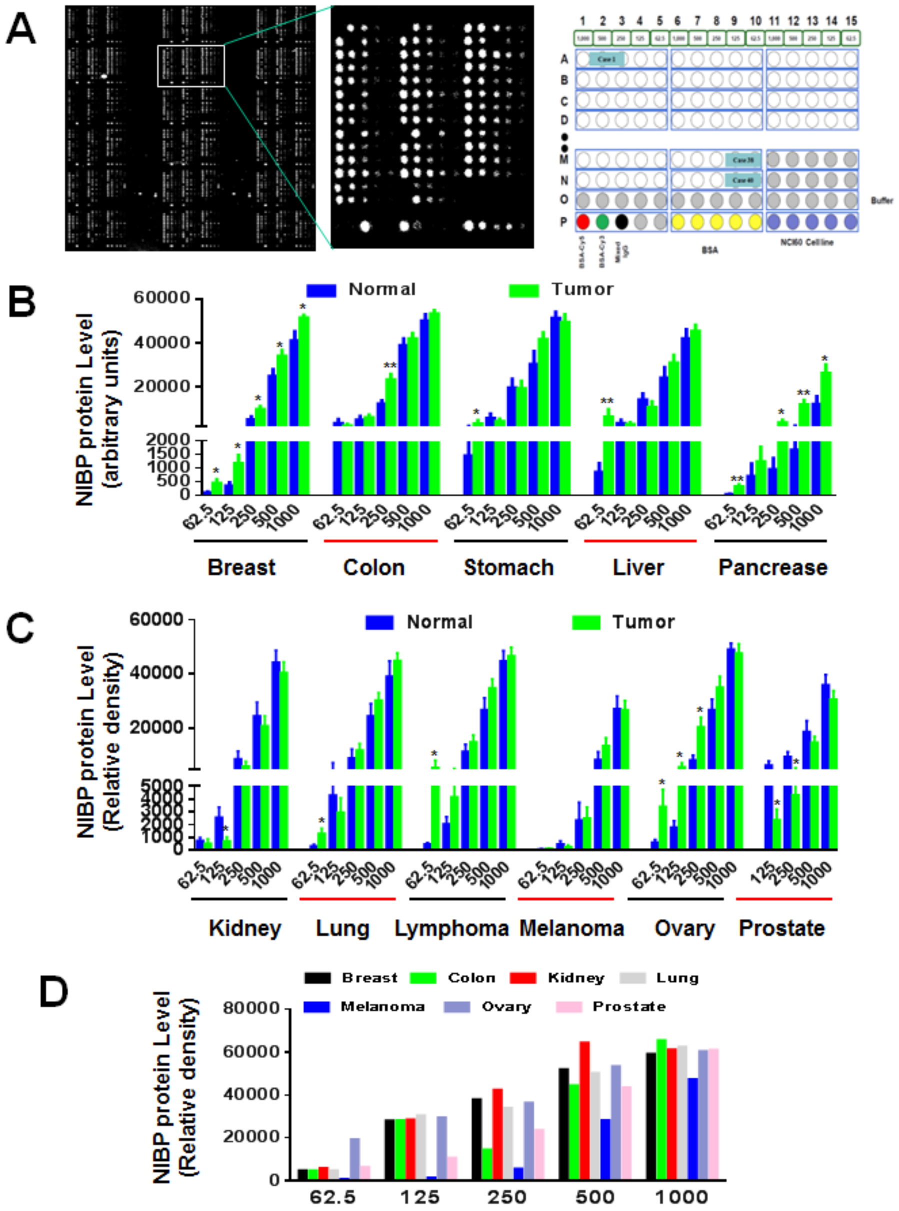

Figure S4: NIBP protein expression in a ProteoScan Cancer Lysate Array (PA100002, OriGene) containing 431 protein lysates of normal and cancer specimens derived from 11 indicated tissues. The array was probed with rabbit anti-NIBP antibody followed by IRDye 800CW-conjugated anti-rabbit secondary antibody and scanned using the Odyssey Infrared Imaging System. (A) A representative layout showing one sub-array with the configuration of 15 x 16 spots containing triplicated samples each at 5 concentration (62.5, 125, 250, 500 and 1,000 µg/ml). (B, C) Relative NIBP protein expression in normal and tumor tissue lysates from 11 tissues. * $p<0.05$ and ** $P<0.01$ indicates significant changes between the tumor and corresponding normal tissues by Student's *t* test. (D) Relative expression levels of NIBP in 7 cancer cell lines corresponding to indicated tissues (MCF7, HCT116, RXF-631D1, HOP-62, SK-MEL-5, OVCAR-5, PC-3).

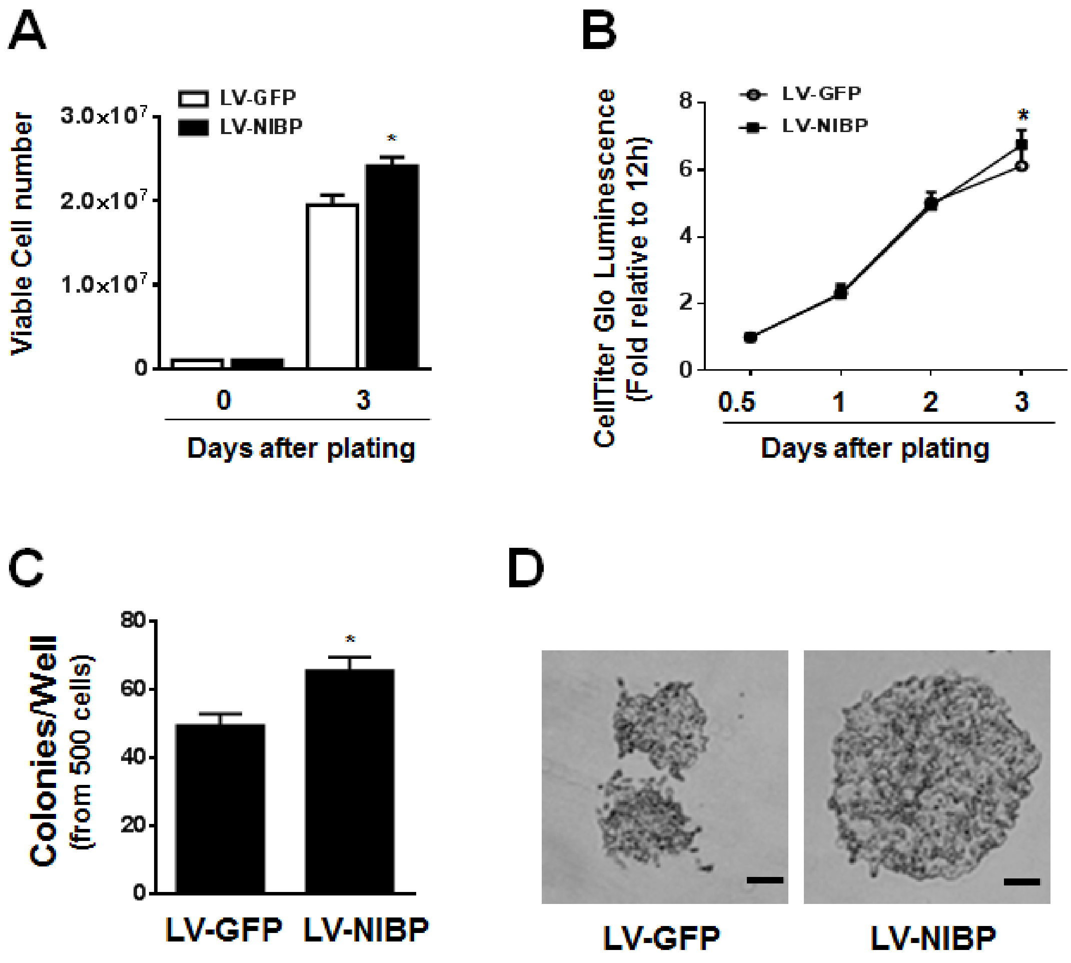

Figure S5: Lentivirus (LV)-mediated NIBP overexpression promotes the cell growth/proliferation and colony formation of colonic cancer cells. HCT116 Cells were infected with LV-NIBP or control LV-GFP followed by hemocytometry (A), Cell-Titer Glo luminescence viability assay (B) and anchorage-dependent colony formation assay (C, D). The data represent mean ± SD of 3-5 independent experiments. * $p<0.05$ indicates significant change in the NIBP overexpression group as compared with the LV-GFP control group. Scale bar = 200 μm.
